# Shaping the Microbial Landscape: Parasitoid-Driven Modifications of *Bactrocera dorsalis* Microbiota

**DOI:** 10.1007/s00248-024-02393-0

**Published:** 2024-06-03

**Authors:** Rehemah Gwokyalya, Jeremy K. Herren, Christopher W. Weldon, Shepard Ndlela, Joseph Gichuhi, Nehemiah Ongeso, Anne W. Wairimu, Sunday Ekesi, Samira A. Mohamed

**Affiliations:** 1 International Centre of Insect Physiology and Ecology, P.O. Box 30772-00100, Nairobi, Kenya; 2 Department of Zoology and Entomology, University of Pretoria, Private Bag X20, Pretoria, South Africa

**Keywords:** Gut microbiome, *Diachasmimorpha longicaudata*, *Psyttalia cosyrae*, Parasitisation, Bacteria, Tephritid, Fruit fly

## Abstract

Koinobiont endoparasitoids regulate the physiology of their hosts through altering host immuno-metabolic responses, processes which function in tandem to shape the composition of the microbiota of these hosts. Here, we employed 16S rRNA and ITS amplicon sequencing to investigate whether parasitization by the parasitoid wasps, *Diachasmimorpha longicaudata* (Ashmaed) (Hymenoptera: Braconidae) and *Psyttalia cosyrae* (Wilkinson) (Hymenoptera: Braconidae), induces gut dysbiosis and differentially alter the gut microbial (bacteria and fungi) communities of an important horticultural pest, *Bactrocera dorsalis* (Hendel) (Diptera: Tephritidae). We further investigated the composition of bacterial communities of adult *D. longicaudata* and *P. cosyrae* to ascertain whether the adult parasitoids and parasitized host larvae share microbial taxa through transmission. We demonstrated that parasitism by *D*. *longicaudata* induced significant gut perturbations, resulting in the colonization and increased relative abundance of pathogenic gut bacteria. Some pathogenic bacteria like *Stenotrophomonas* and *Morganella* were detected in both the guts of *D. longicaudata*-parasitized *B. dorsalis* larvae and adult *D. longicaudata* wasps, suggesting a horizontal transfer of microbes from the parasitoid to the host. The bacterial community of *P*. *cosyrae* adult wasps was dominated by *Arsenophonus nasoniae*, whereas that of *D. longicaudata* adults was dominated by *Paucibater* spp. and *Pseudomonas* spp. Parasitization by either parasitoid wasp was associated with an overall reduction in fungal diversity and evenness. These findings indicate that unlike *P. cosyrae* which is avirulent to *B. dorsalis*, parasitization by *D*. *longicaudata* induces shifts in the gut bacteriome of *B*. *dorsalis* larvae to a pathobiont-dominated community. This mechanism possibly enhances its virulence against the pest, further supporting its candidacy as an effective biocontrol agent of this frugivorous tephritid fruit fly pest.

**Supplementary Information:**

The online version contains supplementary material available at 10.1007/s00248-024-02393-0.

## Introduction

Microbes have emerged as key drivers of host-natural enemy interactions of several horticultural insect pests, often shaping the evolutionary aspects of these bi-partite models. This is primarily through regulating semiochemical production and release, nutrient metabolism, immune function, development, adult size, and other host fitness traits [1–4]. Hence, understanding the dynamics and regulatory patterns of microbial communities is crucial to deciphering the ecological functioning of insect pests, especially with their natural enemies, such as parasitoid wasps.

Insect microbial homeostasis is regulated by several factors including host diet, immune function, sex, developmental stage, geographical location, and biotic stressors including parasitoids [5–10]. Parasitoids are insects that lay eggs in or on other insects (the hosts) eventually killing the hosts. The immature stages of some parasitoid wasps, the endoparasitoids, develop inside and entirely depend on their hosts for sustenance [8, 11]. As such, they evolved mechanisms to tightly regulate their hosts’ immune defenses and nutrient utilization as they develop and feed on their host tissues or hemolymph [8, 12].

Consequently, depending on their nutritional requirements and immunomodulation tactics, parasitoids could alter the microbiota of their hosts to a community that is nutritionally beneficial and/or synergistic with their immunoregulatory strategies. In turn, shifts in microbial composition impact host-parasitoid interactions, posing significant consequences for host-microbe-parasitoid evolution [13]. For instance, parasitism may favor the proliferation of opportunistic pathogenic microbes that weaken host immune defenses [10, 13, 14], facilitating the development of the immature parasitoid.

Conversely, parasitism could trigger the proliferation of defensive microbes which, via resource competition and/or upregulation of host immune defense, protect the host against the invading parasitoid [2, 4, 14]. Parasitoids may also transfer their own microbes to their host insects such that the hosts acquire entirely new microbe(s) [1, 8, 15] that can contribute to the immune and metabolic homeostasis of the hosts as well as their interactions with other trophic levels [1]. Moreover, when injected into the host, parasitoid viral symbionts disrupt host immune and hormonal functioning and development among others, inevitably shifting the resident microbiome of the parasitized host [11]. These parasitoid-mediated modulations of host physiology and microbial community strongly impact gut microbial homeostasis in the host [8]. However, far less is known about host gut microbial homeostasis in insects as a function of parasitism, leaving a paucity of knowledge about the mechanisms underlying insect-microbe-parasitoid interactions.

*Bactrocera dorsalis* (Hendel) (Diptera: Tephritidae) is a major pest of global horticultural production [16]. This pest is associated with a diverse microbial community [3, 17–19] which contributes to its eco-physiological roles such as oviposition behavior, development, immunity, nutritional profiles [3, 20–22], and defense against parasitoids of this pest [23].

The larval-prepupal parasitoids, *Diachasmimorpha longicaudata* (Ashmead) and *Psyttalia cosyrae* (Wilkinson) (both Hymenoptera: Braconidae), have been investigated for use as biocontrol agents of *B. dorsalis*. Notably, they exhibit distinct virulence against this pest with the former successfully parasitizing *B. dorsalis* and the latter failing to develop [24, 25]. This is despite the similarities in their development styles as koinobiont endoparasitoids known to manipulate their hosts’ immunity and nutrient metabolism for the successful development of their immatures. This disparity in virulence has largely been attributed to host-parasitoid evolutionary history and to some extent, differences in *B. dorsalis* immune responses to the two parasitoids [24, 26]. Moreover, viruses including the entomopox virus, DlEPV [27, 28], a rabdho virus, DlRhv [29], and a rod-shaped virus [30] are reported to be associated with *D*. *longicaudata*. The DlEPV has been shown to replicate inside its host [31] and to alter host immune responses by inducing cytopathic effects in host hemocytes [32]. With regard to *Psyttalia cosyrae*, nothing is known about its venom constituents and its host regulatory mechanisms. However, studies show that it does not successfully develop in hosts like *B*. *dorsalis* due to its inability to overcome the immune defenses of this pest [24, 25].

Since these parasitoids exhibit varying immunoregulatory mechanisms and most certainly, distinct parasitism abilities in *B. dorsalis*, it is possible that they differentially alter the structure and diversity of the microbial communities of this frugivorous pest. However, the impact of these parasitoids on the composition of the gut microbiota of *B. dorsalis* remains unexplored. Hence, in this study, we investigated the hypothesis that parasitization by the virulent parasitoid wasp, *D. longicaudata* and its avirulent counterpart, *P. cosyrae* differentially alters the composition of the gut microbiota of *B. dorsalis*. We further explored the bacterial communities of these parasitoids to unravel the interdependence between the host and parasitoid microbiomes to investigate possible horizontal transmission of bacteria taxa between the parasitoids and their hosts, *B*. *dorsalis* larvae.

## Methods

### *Bactrocera Dorsalis* and Parasitoid Rearing

*Bactrocera dorsalis* used in this study were obtained from a sample of infested mangoes collected from the field in Embu (S 0° 28′ 56.6″E 37° 34′ 55.5″), Eastern Kenya, and incubated at the insectary of the International Centre of Insect Physiology and Ecology (*icipe*). The emerged flies were identified and a culture of *B. dorsalis* was initiated and maintained as previously described [33] on a larval liquid diet [34] modified by excluding streptomycin and nipagin. The rearing conditions were set at a temperature range of 25–27 °C, 60–70% relative humidity, and a 12:12 day:night photoperiod. Enclosed adult flies were fed on yeast and water ad libitum [25]. *Bactrocera dorsalis* flies were maintained for three generations prior the experiments.

The parasitoids, *D. longicaudata* (183rd generation) and *P. cosyrae* (177th generation) used in this study, were also reared in the insectary at *icipe* under the same conditions described above. *Psyttalia cosyrae* was reared on a laboratory colony of *Ceratitis cosyra* Walker (Diptera: Tephritidae), while *D. longicaudata* was reared on *B. dorsalis* as described by Mohamed et al. [35] and Mohamed et al. [26], respectively.

### Exposure of *B. dorsalis* Larvae to Parasitoids

Freshly cut mango domes (mango cut into half and seed and pulp scooped out) were exposed to gravid 7-day old *B. dorsalis* females, as an oviposition substrate. Subsequently, the eggs were harvested from the mango domes and reared on a liquid diet as described above. Using soft forceps, a set of 100 2nd instar larvae were randomly selected and transferred to larval oviposition units containing a semi-solid carrot diet [34] modified as in Gwokyalya et al. [23] by omitting nipagin and streptomycin. The oviposition units containing the larvae were offered to either *D. longicaudata* (*n* = 10 7-day old females) or *P. cosyrae* (10 7-day old females) held in separate Perspex cages (12 × 12 × 12 cm). The females of the former were allowed to oviposit for 2 h, while for the latter, 6 h. Thereafter, larvae were retrieved from the oviposition units and transferred to carrot diet held in 2-L transparent lunch boxes (18 × 11 × 15 cm) covered with a cotton mesh for subsequent bioassays.

### Dissection of *B. dorsalis, D. longicaudata*, and *P. cosyrae* Guts

Forty-eight hours after exposure to the parasitoids, *B*. *dorsalis* larvae were dissected under a stereomicroscope (Zeiss Stemi 508, Zeiss, Oberkochen, Germany), to ascertain parasitism (presence of a parasitoid larva and/or egg). Once parasitism was confirmed, the guts of the larvae were extracted as described [3]. Briefly, the larvae were surface sterilized in 2% sodium hypochlorite solution, 70% ethanol, and in distilled water, sequentially. The sterilized larvae were transferred to a drop of phosphate-buffered saline (PBS) solution on a sterile Petri dish. Then the larval guts were dissected under a stereomicroscope and transferred to autoclaved 1.5-mL Eppendorf tubes. From each biological replicate parasitized by each parasitoid species, a total of 10 larval guts were pooled in one tube and this was replicated six times. Guts from unexposed early 3rd instar *B. dorsalis* larvae were extracted in the same manner described for their parasitized counterparts and used as a control.

In a separate bioassay, guts of 3-day old female wasps of *D. longicaudata* and *P. cosyrae* reared on their respective host insect were extracted using the same procedure described for the parasitized larvae. Guts from 10 adult female parasitoids of each species were separately pooled in one Eppendorf tube for consequent bacteriome analyses. The guts of *B. dorsalis* larvae as well as that of the two parasitoid species were stored at – 20 °C until DNA extraction.

### DNA Extraction and Sequencing

Genomic DNA (gDNA) for microbiome analysis (bacteria and fungi) was extracted from the guts of *B*. *dorsalis* larvae, *D. longicaudata*, and *P. cosyrae* using the Bioline genomic DNA kit (Meridian Biosciences, Cincinnati, OH, USA) following the manufacturer’s instructions. The extracted DNA was quality checked using a NanoDrop spectrophotometer (NanoDrop™ 2000, Thermo Scientific, DE, USA). Samples with DNA concentrations of at least 50 ng were shipped for sequencing using the Illumina MiSeq, 2 × 300 bp amplicon sequencing at Macrogen Inc. (Seoul, South Korea). Bacterial amplicon sequencing was done using primers targeting the V3–V4 region (518F CCAGCAGCCGCGGTAATACG, 800R TACCAGGGTATCTAATCC), whereas fungal sequencing was done using primers targeting the ITS2 region (ITS3 GCATCGATGAAGAACGCAGC, ITS4 TCCTCCGCTTATTGATATGC) for *B. dorsalis* larval guts only.

### Bioinformatic Analysis

Metagenomic analysis of the demultiplexed raw reads was done using the DADA2 pipeline (version 1.18.0) [36] in R studio (version 4.2.2) [37]. Firstly, reads were trimmed using the following parameters: filterAndTrim (250, 230) function, truncQ = 2, maxEE = 2, 5, rm.phix set to TRUE, and maxN set to 0. The resultant reads were dereplicated and merged, after which chimeric sequences were removed, generating amplicon sequence variants (ASVs). Taxonomy assignment of the bacterial and fungal ASVs was done using the Silva 138 [38] and UNITE general FASTA release for fungi from the UNITE (version 16.10.2022) [39] databases, respectively. The generated ASV count matrix, taxa assignment tables, and the sample metadata file were merged into a phyloseq object using the phyloseq package (version 1.34) [40].

The relative abundance of the bacterial and fungal communities was analyzed based on the relative abundances of the genera and species using the metagMisc package (v 0.04) [41] and visualized as stacked bar plots. To assess species alpha and beta diversity of the 16S and ITS communities, the ASV read counts were rarefied to assess adequate sampling of the microbial communities. The rarefied reads were then used to compute microbial alpha diversity, which was inferred from and depicted in the Chao1, Pielou, and Shannon indices using the MicrobiotaProcess package (v1.9.3) [42]. Beta diversity analysis was conducted using the weighted Uni-frac index and the dissimilarity among the treatments was depicted in a principal coordinates analysis (PCoA). Core taxa (defined as ASVs present in 90% of the samples at a 75% prevalence) shared between the *B*. *dorsalis* larval treatments were identified and visualized as Venn diagrams using the microbiome [43] and eulerr [44] packages.

The interaction between the bacterial community (genus level) and the *B. dorsalis* (control, *D*. *longicaudata* parasitized, and *P. cosyrae* parasitized) larvae as well as the parasitoids was assessed using the Bipartite package (version 2.18) [45]. A parasitoid/*B*. *dorsalis*-bacteria matrix integrating the relative abundance data (quantitative) of the bacteria was used to generate a bipartite web plot linking the nodes of *B*. *dorsalis* larvae as well as those of the parasitoids to the bacteria genera detected in each group. We proceeded to analyze the overall web topography indices from which we inferred the network C. score and the degree of nestedness. However, since nestedness is subject to biases arising from the matrix size, we compared the nestedness values from the network-level output to null models using 1000 simulated replicates. Network modularity was analyzed and visualized in R (version 4.2.1).

### Statistical Analysis

To investigate the impact of parasitization on the abundance of bacterial genera in *B. dorsalis*, differential abundance analysis of bacterial ASVs was done using the negative binomial log-linear model in DESeq2 [46]. Based on the outcome of DESeq2, differentially abundant ASVs (*P* < 0.05) were selected for further pairwise comparison using total sum scaling log2 linear regression analysis at the genus level in microViz package [47]. For alpha diversity, significant differences recorded in any of the indices were further investigated using Kruskal-Wallis pairwise comparison test to ascertain the differences in microbial diversity due to parasitization by either parasitoid species. The effect of parasitization on microbiome beta diversity was tested by permutational multivariate analysis of variance (PERMANOVA) [48] using the adonis2 function in the vegan package (version 2.5.7) [49]. Additionally, a beta-dispersion test was conducted to infer statistical differences between the variances of the microbial communities of *B*. *dorsalis* larval treatments (betadisper function in vegan). Where significant differences were identified, Tukey’s “honest significant difference” (HSD) post-hoc tests were performed to identify significant differences between the *B*. *dorsalis* larval treatments.

## Results

### Effect of Parasitization on the Bacterial Communities of *B. dorsalis*

Out of 3,278,394 reads (average per sample 182,133 reads, min 70,200, max 283,610 reads) we recorded 3021 bacterial ASVs assigned to 18 phyla, 143 families, and 321 genera. Overall, the main phyla were Pseudomonadota (66.93%) and Bacillota (21.13%). Additional data is given in Online Resource 1.

**Fig. 1:**
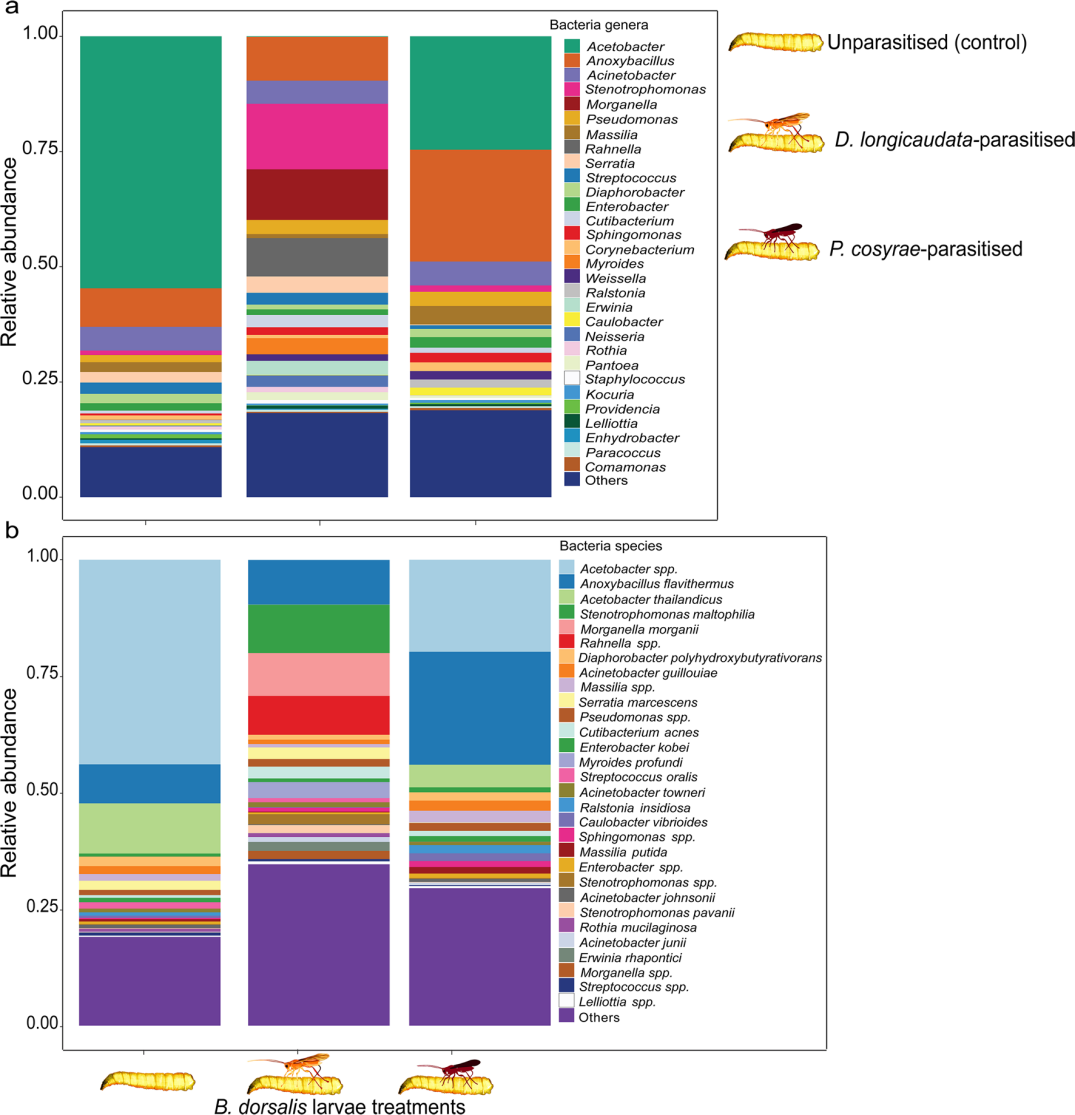
Relative abundances of the top 30 gut bacteria of *Bactrocera dorsalis* larvae at the genus (**a**) and species (**b**) levels post-parasitization by *Diachasmimorpha longicaudata* and *Psyttalia cosyrae*

At the genus and species levels, the bacterial community of the unexposed controls mainly comprised *Acetobacter* (55.3%, mainly *Acetobacter thailandicus*), *Anoxybacillus* (8.4%, especially *Anoxybacillus flavithermus*), and *Acinetobacter* (5.0%, mainly *Acinetobacter guillouiae*) genera (Fig. 1a, b). Exposure to parasitoids shifted the relative abundance of the gut bacterial communities of *B. dorsalis*, especially among *D. longicaudata*-parasitized larvae, which predominantly comprised *Stenotrophomonas*, *Anoxybacillus*, and *Morganella* genera at 14.4%, 9.6%, and 11.1%, respectively (Fig. 1a). Also, *D. longicaudata*-parasitized larvae harbored unique bacterial species: *Erwinia rhaphontici* (1.9%), *Myroides profundi* (3.5%), *Rahnella spp.* (8.3%), and *Morganella morganii* (9.2%), which were present in neither the unparasitized larvae nor in those parasitized by *P*. *cosyrae* (Fig. 1b).

The gut bacterial community of larvae parasitized by *P. cosyrae* was more similar to that of the unparasitized larvae and was largely composed of species belonging to *Acetobacter* (24.9%), *Anoxybacillus* (24.6%), *Acinetobacter* (4.9%), and *Masillia* (4.1%) genera (Fig. 1a, b). Bacteria species belonging to *Pantoea* and *Weisella* genera were only found in the guts of larvae parasitized by both parasitoids but not in those of the un-parasitized controls (Fig. 1a, b). Additional data is provided in Online Resource 2.

**Fig. 2:**
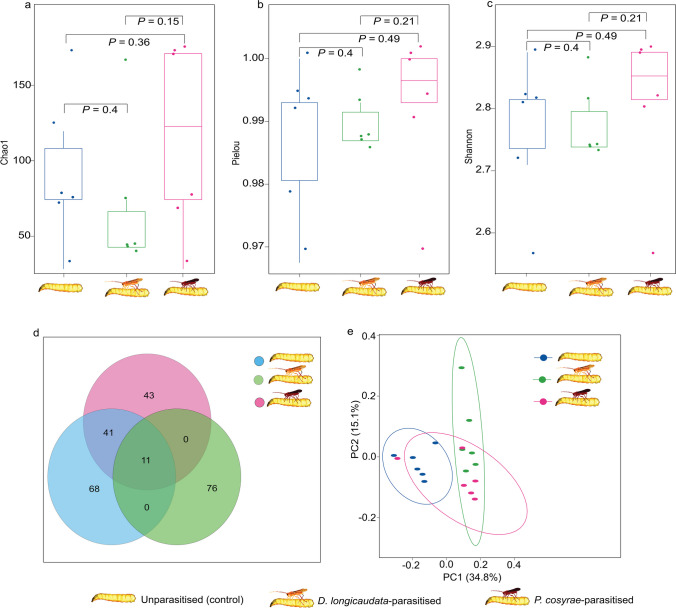
Diversity of the bacteria of *Bactrocera dorsalis* larvae guts post-parasitization by *Diachasmimorpha longicaudata* and *Psyttalia cosyrae*. Alpha diversity as depicted by the (**a**) Chao1’s richness, (**b**) Pielou’s evenness, and (**c**) Shannon’s diversity indices. The numbers on the boxplots are pairwise comparisons between the respective *B. dorsalis* larval treatments (treatments are statistically significant if *P* < 0.05, Kruskal-Wallis test). Venn diagram indicating the number of shared core bacterial amplicon sequence variants in the gut of the unparasitized larvae and those parasitized by the wasps (**d**) and beta diversity as a function of the principal component analysis based on the Bray-Curtis (**e**)

Bray-Curtis dissimilarity index-based PCoA results depicted a distinct clustering of the unparasitized larvae from the *D*. *longicaudata*-parasitized larvae and shared clustering of the microbiota of *P. cosyrae*-parasitized larvae with these two treatments (Fig. 2e). *Psyttalia cosyrae* and *D. longicaudata*-parasitized larvae had the lowest and highest numbers of core ASVs, respectively (Fig. 2d). *Psyttalia cosyrae*-parasitized larvae and the unparasitized controls shared many bacterial ASVs (almost as many as the core ASVs in the individual). *Diachasmimorpha longicaudata*-parasitized larvae shared no ASVs with the other two treatments individually but had 11 ASVs which were common among all the treatments (Fig. 2d).

The PERMANOVA analysis identified significant compositional dissimilarity in the bacterial communities across the *B*. *dorsalis* larval treatments post-parasitization (*P* < 0.001) and revealed that parasitization explains 27% of this variance (*R*^2^ = 0.270). The beta dispersion analysis revealed homogenous dispersion across the *B*. *dorsalis* larval treatments (ANOVA, *P* = 0.148, *F* = 2.173, df = 2). There were neither significant differences between the bacterial community of the control larvae and that of *P*. *cosyrae*-parasitized larvae (Tukey’s HSD post hoc *P adj* = 0.385) nor between the controls and the *D*. *longicaudata*-parasitized larvae (Tukey’s HSD post hoc *P adj* = 0.186) nor between the bacterial communities of larvae parasitized by either parasitoid (Tukey’s HSD post hoc *P adj* = 0.875). There were no significant differences in the bacterial alpha diversity due to parasitization as revealed by the Shannon, Chao 1, and Pielou indices.

### Influence of Parasitization on the Differential Abundance of Bacterial Communities of *B. dorsalis*

**Fig. 3:**
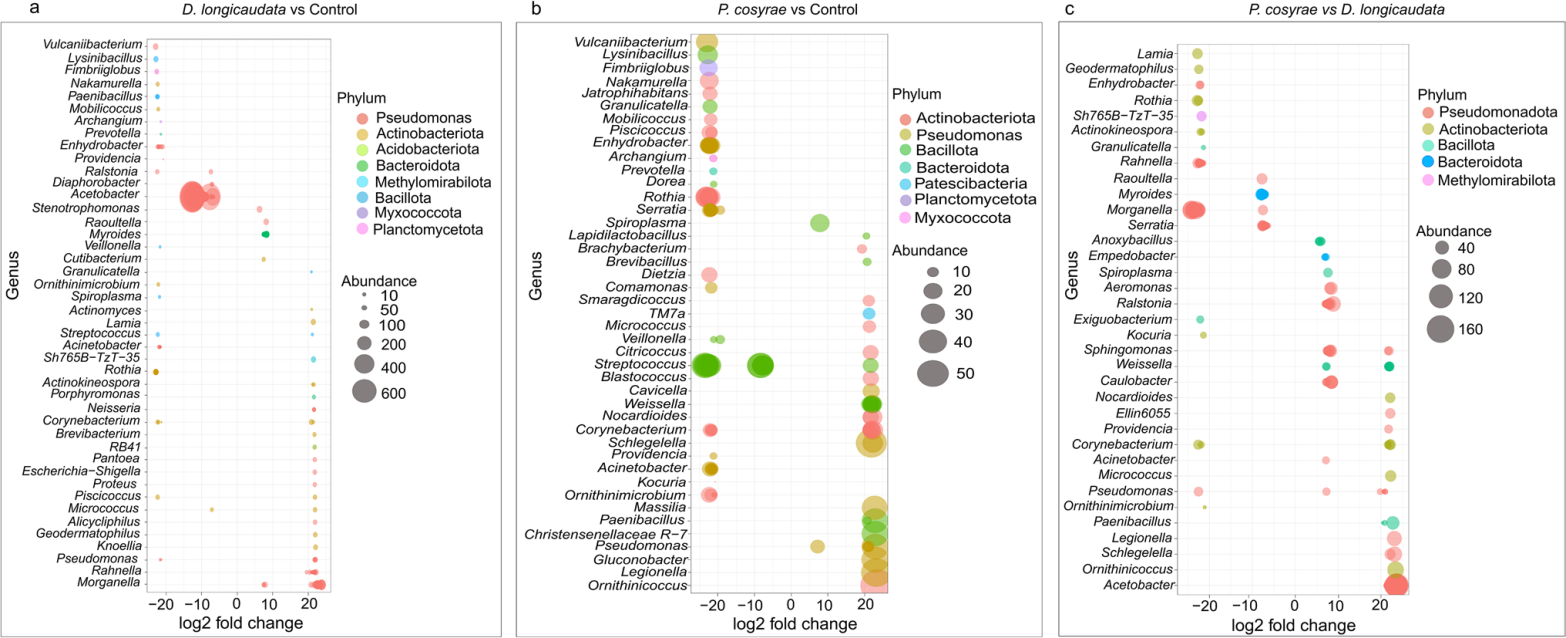
Bubble plot showing taxon level effect of parasitization on *B*. *dorsalis* bacterial community. Differentially abundant ASVs in (**a**) *Diachasmimorpha longicaudata*-parasitized larvae compared to the control, (**b**) *Psyttalia cosyrae*-parasitized larvae compared to the control, and (**c**) *P*. *cosyrae*-parasitized larvae compared to those parasitized by *D*. *longicaudata*. Each bubble represents an individual ASV. ASVs with a log2-fold change significantly different from 0 (padj. < 0.05) and classified at the genus level are shown. In the *D*. *longicaudata* vs. control and the *P. cosyrae* vs. *D*. *longicaudata* comparisons, the most abundant 250 ASVs fulfilling these criteria are shown, whereas in the *P*. *cosyrae* vs. control comparison, the topmost 150 ASVs are shown. The size of each bubble indicates the abundance of the individual ASVs in the respective *B*. *dorsalis* larvae comparison. Complete indicator ASV lists are provided in Online Resources 3, 4, and 6

By identifying differentially abundant bacterial ASVs from the relative abundance analysis, we were able to detect individual taxa that were significantly influenced by parasitization in *B*. *dorsalis* larvae. Parasitization by *D*. *longicaudata* significantly influenced the abundance of several genera (461 ASVs; additional data is provided in Online Resource 3). Notably, the relative abundances of some genera like *Morganella*, *Stenotrophomonas*, *Pantoea*, and *Serratia* significantly increased, whereas the relative abundance of *Acetobacter* reduced relative to the unparasitized control (Fig. 3a; additional data are given in Online Resources 3 and 4). On the other hand, the relative abundances of 340 bacteria ASVs were significantly affected post-parasitization by *P*. *cosyrae*. Relative abundances of bacteria ASVs belonging to genera such as *Pseudomonas*, *Weissella*, and *Massilia* were higher while those of *Streptococcus* and *Serratia* decreased in larvae parasitized by this wasp compared to the control (Fig. 3b; additional data are given in Online Resources 3 and 5). Comparing the relative abundances of the bacterial ASVs of *B*. *dorsalis* larvae parasitized by either parasitoid revealed significant differences in 526 ASVs. The relative abundances of ASVs belonging to bacteria genera like *Acetobacter*, *Anoxybacillus*, *Providencia*, and *Weissella* were higher, whereas those of bacteria genera such as *Serratia*, *Morganella*, *Myroides*, and *Rahnella* were significantly lower in *P*. *cosyrae*-parasitized larvae compared to those parasitized by *D*. *longicaudata* (Fig. 3c; additional data are given in Online Resources 3 and 6).

### Variation in the Bacterial communities of Adult Parasitoids

**Fig. 4:**
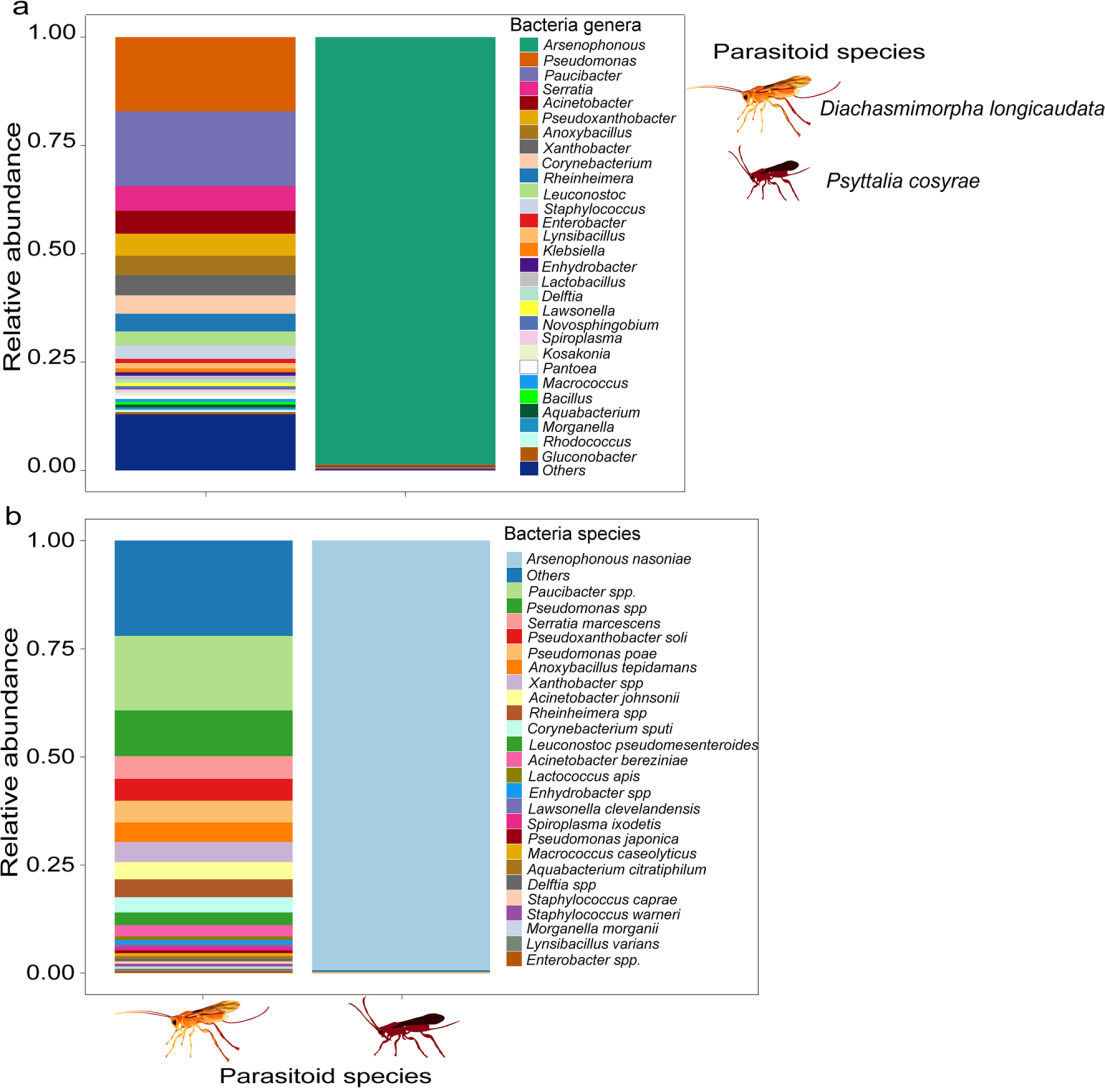
The relative abundance of the top 30 gut bacteria of adult female *Diachasmimorpha longicaudata* and *Psyttalia cosyrae* wasps at the genus (**a**) and species (**b**) levels

There were notable variations in the relative abundances of the bacterial communities of the two parasitoid species, *D*. *longicaudata* and *P. cosyrae* (Fig. 4a, b). The bacterial community of *D. longicaudata* was dominated by *Paucibacter spp.* (17.8%), *Pseudomonas spp*. (11.3%), *Serratia marcescens* (5.8%), *Anoxybacillus tepidamans* (5.0%), and *Acinetobacter johnsonii* (3.7%). On the other hand, the microbiota of *P*. *cosyrae* mainly comprised *Arsenophonus nasoniae* (99.9%) (Fig. 4a, b; additional data are given in Online Resource 7).

### Bacteria-*B. dorsalis* Larvae/Parasitoid Network Analysis

**Fig. 5:**
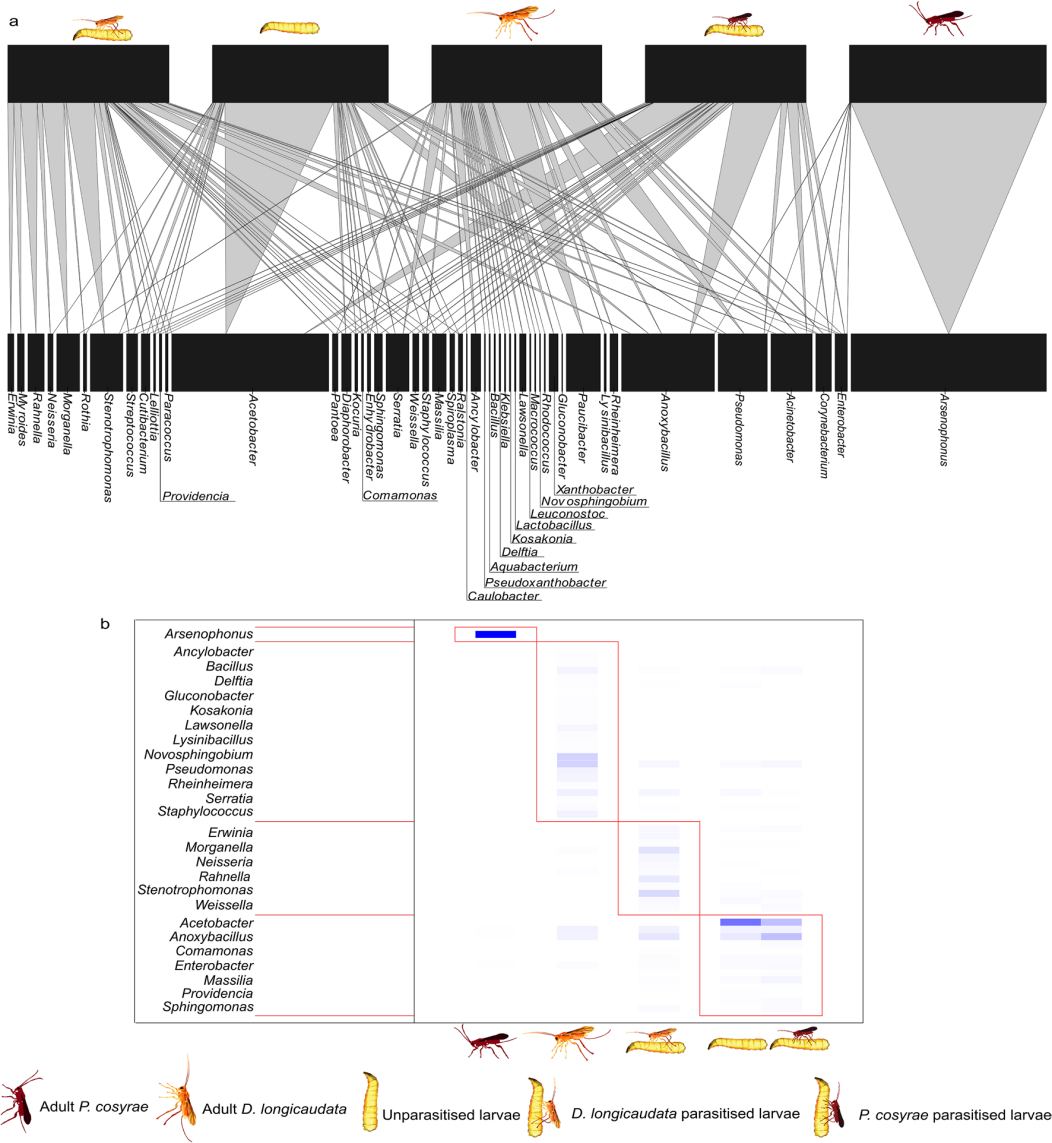
Bipartite network of bacterial communities-*Bactrocera dorsalis* and parasitoid (*Diachasmimorpha longicaudata* and *Psyttalia cosyrae*) associations. (**a**) Bipartite graph showing patterns of interaction between bacteria genera present across the different *B. dorsalis* larval groups and the adult parasitoids. The upper nodes represent *B. dorsalis* larvae and parasitoid species while the lower nodes represent the bacteria genera. The length of each node is scaled to the total number of interactions for each object (i.e., the bacteria genus, *B. dorsalis* larval group, or parasitoid species). The links (gray lines) connecting two nodes represent the interaction between the bacterial genera and the *B*. *dorsalis* larvae or parasitoid species. The widths of the links are scaled to the number of interactions between each pair of nodes (each bacteria genus and the respective *B*. *dorsalis* larval group/parasitoid species). (**b**) Modular bipartite matrix of identified modules based on the bipartite network analysis of shared bacteria genera among the *B. dorsalis* larvae and parasitoid species. The intensity of the color in each box indicates the number of interactions identified between the modules

The bipartite network revealed complex interactions between the bacterial communities and the respective *B*. *dorsalis* larvae and parasitoid species. Some bacteria genera, i.e., *Enterobacter*, *Anoxybacillus*, and *Acinetobacter*, were present in majority of *B*. *dorsalis* larval groups and the adult parasitoids, whereas others such as *Arsenophonous*, *Morganella*, *Neisseria*, and *Rahnella* were only present in one or two *B*. *dorsalis* larval groups or in the parasitoids only. *Psyttalia cosyrae* adults had the least number of associations with bacteria genera, whereas *B*. *dorsalis larvae* parasitized by *D*. *longicaudata* had the highest number of interactions (Fig. 5a). The interaction matrix showed high levels of nestedness and modularity across the individual larvae/parasitoid-bacteria networks (nestedness = 27.348, modularity *Q* score = 0.571, C. score 0.256, *P* = 0.016, Fig. 5b). The unparasitized and *P*. *cosyrae*-parasitized *B. dorsalis* larvae clustered together, whereas *P*. *cosyrae* adults, *D*. *longicaudata* adults, and *D*. *longicaudata*-parasitized *B*. *dorsalis* larvae clustered independently (Fig. 5b).

### Effect of Parasitization on the Fungal Communities of *B. dorsalis* Larvae

**Fig. 6:**
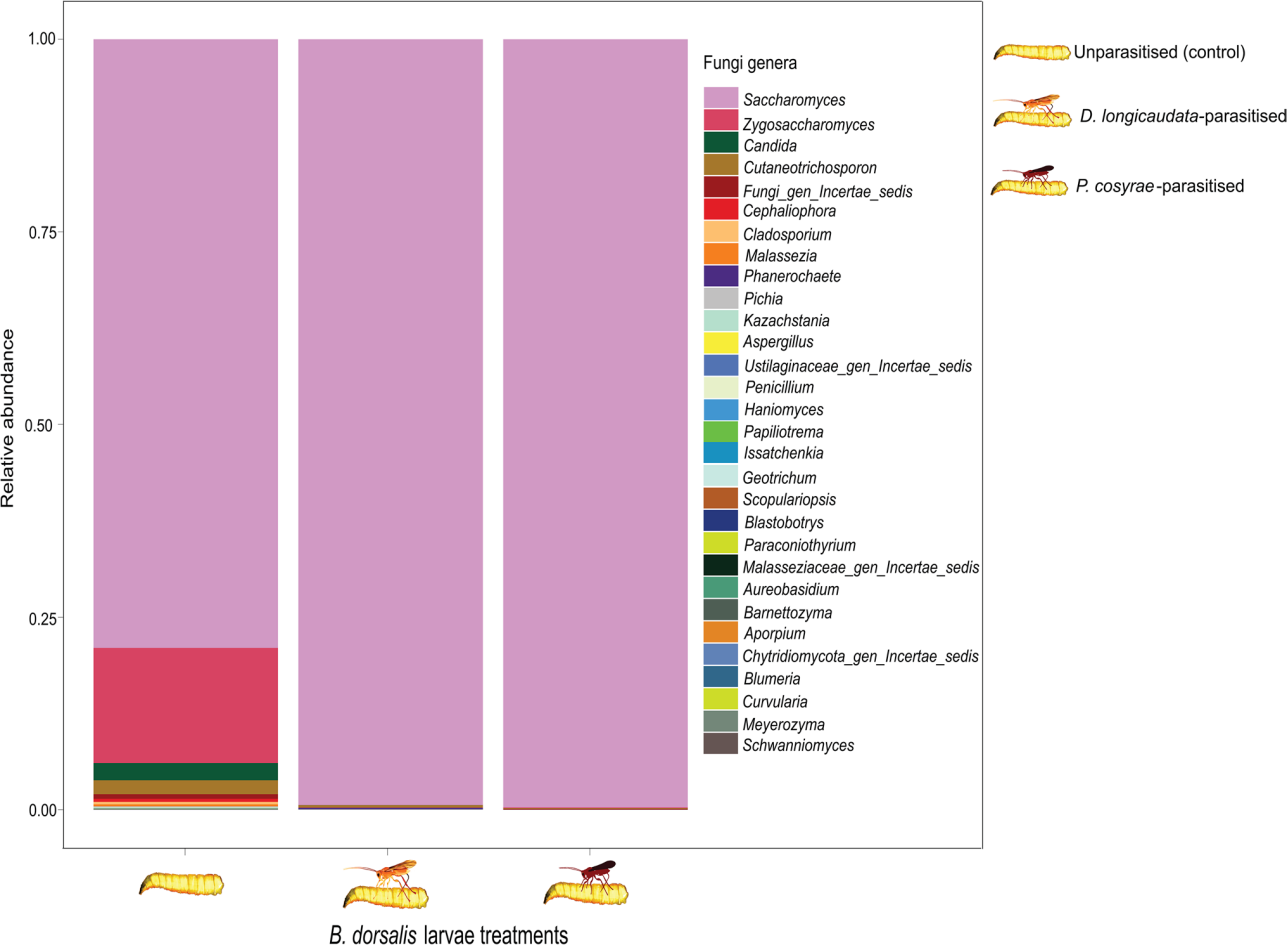
Relative abundance of the gut fungal genera of *Bactrocera dorsalis* larvae post-parasitization by *Diachasmimorpha* *longicaudata* and *Psyttalia* *cosyrae*

Out of 3,991,032 reads (average per sample 221,724 reads, min 173,586, max 271,674 reads), we recorded 123 fungal ASVs belonging to 3 phyla, 24 families, and 86 genera. Overall, Ascomycota (98.9%) was the dominant phylum followed by Basidiomycota (0.9%) and unclassified fungi (0.2%) as illustrated in Online Resource 8). The unexposed larvae were largely composed of *Saccharomyces* (79.0%), *Zygosacchharomyces* (14.9%), and *Candida* (2.3%) genera (Fig. 6). However, this compositional trend changed after parasitization; the relative abundance of all fungal genera except *Saccharomyces* reduced after parasitization by either parasitoid species (Fig. 6; additional data is provided in Online Resource 9).

**Fig. 7:**
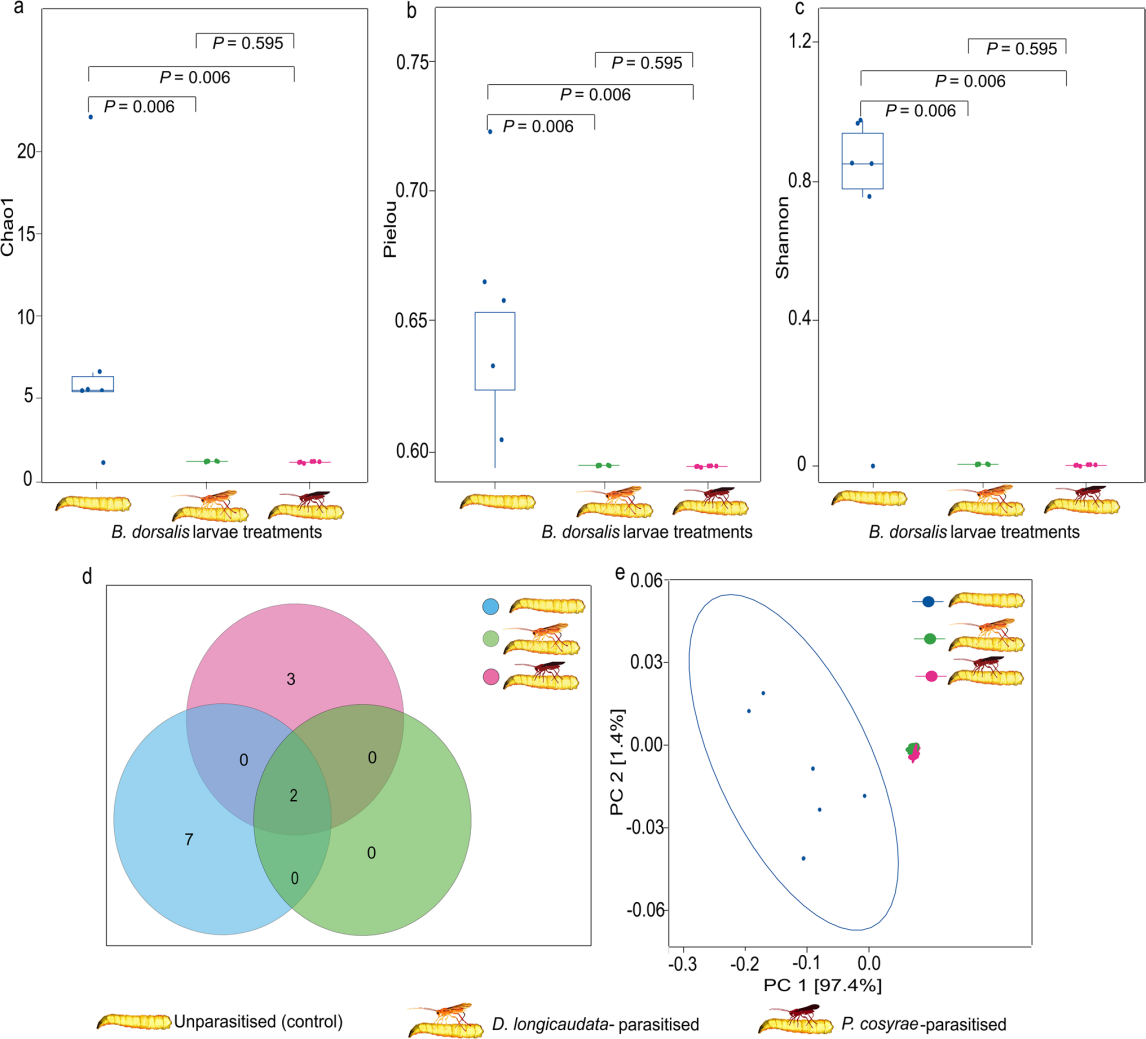
Diversity of the fungi of *Bactrocera dorsalis* larval guts post-parasitization by *Diachasmimorpha longicaudata* and *Psyttalia cosyrae*. Fungi alpha diversity as depicted by the (**a**) Chao1’s richness, (**b**) Pielou’s evenness, and (**c**) Shannon’s diversity indices. Venn diagram comparing the number of shared core fungal amplicon sequence variants in the gut of the unparasitized larvae and those parasitized by the wasps (**d**) and beta diversity of *B. dorsalis* larvae gut fungi as a function of the principal component analysis based on the Bray-Curtis (**e**)

Beta diversity analysis results showed significant differences in the alpha diversity of the gut fungal communities of *B. dorsalis* due to parasitization as indicated by the Chao 1, Pielou, and Shannon indices (Fig. 7a, b, c) that revealed evidently lower diversity and evenness in the fungal communities of the larvae parasitized by either parasitoid species. Furthermore, PCoA results showed distinct clustering of the gut mycobiome of the control larvae while the mycobiomes of those parasitized by either parasitoid clustered together (Fig. 7e). Moreover, the control larvae had the highest number of core fungi, whereas those parasitized by either *D*. *longicaudata* or *P*. *cosyrae* had less core fungal ASVs relative to the control (Fig. 7d).

The PERMANOVA results revealed significant compositional dissimilarity across the *B*. *dorsalis* larval treatments post-parasitization (*P* = 0.001) and revealed that parasitization explained 79.2% of this variance (*R*^2^ = 0.792). However, beta dispersion analysis revealed non-homogenous dispersion across the *B*. *dorsalis* larval treatments (ANOVA, *P* < 0.001, *F* = 14.854, df = 2). Tukey’s HSD post hoc analysis indicated that the mycobiome of the control larvae was significantly different from that of larvae parasitized by either parasitoid (*P adj* = 0.002 and *P adj* < 0.001 for *D*. *longicaudata* and *P*. *cosyrae*, respectively). However, there were no significant differences in the fungal communities of the larvae parasitized by *D*. *longicaudata* and those parasitized by *P*. *cosyrae* (*P adj* = 0.644).

## Discussion

Parasitoids have important consequences for host physiological and ecological function. They influence the interaction of their hosts with their immediate surroundings, including host-microbiota associations [8, 10, 13, 15], potentially shaping host-microbe evolutionary functions. The same could apply in *B. dorsalis*, which is permissive to some parasitoids and not permissive to others [24, 25]. Here we demonstrate that virulent and avirulent parasitoids of *B. dorsalis* alter the gut mycobiome and differentially shape the composition of the gut bacterial communities of this pest.

Similar to earlier microbiota reports from tephritids [3, 5, 6], we found that Pseudomonadota and Bacillota were the most abundant bacterial phyla in *B. dorsalis* larvae. This finding suggests the intimate association between *B. dorsalis* and these phyla, and that they could be contributing to the physiological functioning of this pest such as development and nutrient digestion among others. Earlier studies [3, 20, 23] investigating Pseudomonadota and Bacillota bacterial strains and their roles in the eco-physiological functions of *B. dorsalis* confirm this phenomenon.

*Acetobacter* species, *Acinetobacter* species, as well as *Anoxybacillus* species were highly abundant in the control and the *P*. *cosyrae*-parasitized larvae. Species of the genus *Anoxybacillus* have been linked to the digestion of sugars, cellulose, fats, and proteins [50, 51]. On the other hand, *Acetobacter thailandicus*, like other acetic acid bacteria (see [52] and references therein) could also be involved in the breakdown of sugar in the guts of frugivorous insects such as *B*. *dorsalis*. Therefore, the high relative abundance of these bacteria across these treatments could be due to their metabolic roles in *B*. *dorsalis* larvae. However, *Acetobacter* was detected at low abundances in *D*. *longicaudata*-parasitized larvae, and this could explain the pest control effect on this parasitoid on *B*. *dorsalis* due to lack of sugar metabolism.

Earlier reports suggested that less diverse microbial communities are prone to colonization by pathogenic microbes via reduced niche and nutritional competition as well as suppressed immuno-competence [53]. While the alpha diversity did not change, pathogenic bacteria like *Serratia marcescens* and *Stenotrophomonas maltophilia* were more abundant in *D. longicaudata*-parasitized larval guts. *Serratia marcescens* is a commensal symbiont with mild to no effects on its hosts. However, its proliferation and subsequent translocation to the hemocoel has been shown to be detrimental, rendering it pathogenic rather than commensalistic to its hosts [54, 55]. Indeed, a high load of *S. marcescens* has been reported to induce gut epithelia bloating and thinning in its hosts, which enhances its translocation into the hemocoel and interference with host immune function [55, 56]. As such, this bacterium has been explored for its potential use in the control of arthropod pests and management of disease vectors due to its ability to alter the vector competence of some insects of human health importance such as mosquitoes [56, 57].

*Stenotrophomonas maltophilia* is a common gut bacterium in insects [10, 58] associated with bacteremia in immune-suppressed and immunocompetent systems [59]. We, therefore, postulate that the increased relative abundance of these bacteria could be a result of *D. longicaudata*-induced gut dysbiosis, which shifts the *B*. *dorsalis* larval gut bacteriome to a pathogen-dominated community. Our other studies have found that parasitization by *D. longicaudata* downregulates anti-oxidative genes like glutathione transferases as well as cecropins and lysozyme B, genes responsible for antimicrobial defense in *B. dorsalis* [Gwokyalya et al. unpublished]. It is, therefore, plausible that parasitization by *D. longicaudata* increases the relative abundance of pathogenic gut microbes via suppression of antimicrobial defenses and activation of oxidative stress, interactive processes that advance its virulence against *B. dorsalis*. This finding warrants further investigation of the ecological significance of these bacteria and their implications for parasitoid virulence and pest control.

Previous studies reported increase in specific abundances and/or acquisition of new host gut microbial members after parasitization due to transfer of microbes from the parasitoid to the host, [1, 8, 15]. In this study, we found a similar trend in the parasitized larval guts which comprised *Weisella* and *Pantoea* species, bacteria that were not present in the control larvae. These two bacteria are ubiquitous in the environment and parasitization might have facilitated the introduction into the host larvae and the colonization of the host gut. More interesting, however, was the finding that *M. morganii* was only associated with *D. longicaudata*-parasitized larvae and *D*. *longicaudata*, so it was perhaps transferred from the parasitoid female into the host larvae during parasitization. *Morganella morganii* is an opportunistic bacterium linked to pathogenicity in tephritids [60, 61] and may have an immune-suppressing function in *B*. *dorsalis*.

Contrary to the pathogen-dominated bacterial community recorded in *D*. *longicaudata*-parasitized larvae, parasitization by *P*. *cosyrae* mainly caused significant changes in the abundances of acetic-acid digesting bacteria like *Gluconobacter* and *Acinetobacter*. The disparity in the modulatory mechanisms of *B. dorsalis* larval bacterial community by these two wasps could be attributed to variations in their host regulation strategies. *Psyttalia cosyrae* is unable to surmount the immune defenses of *B. dorsalis* [24, 25] and our preliminary data suggests that parasitization by this wasp increases the expression of antimicrobial peptide (AMP)-related genes such as cecropin and attacin in *B*. *dorsalis* [Gwokyalya et al. unpublished]. Thus, it seems likely that it is these AMPs that suppress the proliferation of the pathogenic microbes leading to increased abundance of the metabolism-aiding microbes. *Diachasmimorpha longicaudata*, on the other hand, injects its symbiotic virus, DlEPV, into its parasitized larvae which markedly disrupts the immune processes [31, 32]. It is likely that the injection of DlEPV contributed to the changes in the composition of the bacterial community of *B*. *dorsalis*. Moreover, the network module results depicted similar module clustering of the control larvae and those parasitized by *P. cosyrae*, suggesting an insignificant impact of this parasitoid on *B. dorsalis* microbiota.

Regarding the parasitoid bacterial communities, the high relative abundance of *Anoxybacillus*, *Acinetobacter*, and *Pseudomonas* bacteria in *D. longicaudata* could be due to their contribution to the nutritional and metabolic needs of this parasitoid. In contrast, the gut bacterial community of *P. cosyrae* was less diverse and was dominated by the bacterium *Arsenophonous nasoniae*, a widely distributed male killing secondary symbiont [62, 63]. Although not reported in other Opine species, the association of *A*. *nasoniae* with *P*. *cosyrae* is not surprising since this symbiont has been reported in other hymenopteran parasitoids [63]. Unexpectedly, we found no association of *A. nasoniae* with *B*. *dorsalis*, a finding that deviates from the theory of shared microbiota due to horizontal symbiont transmission between parasitoids and their hosts [64]. While unclear, it is possible that this could be a selective-association mechanism since *B. dorsalis* and *P. cosyrae* do not share evolutionary history, or that *A. nasoniae* is blocked from colonizing *B. dorsalis* as the parasitoid is encapsulated at the egg stage alongside the parasitoid venom cocktail [24]. These arguments, however, warrant further investigation to unravel the evolutionary aspects, transmission mechanisms, and eco-physiological implications of harboring *A*. *nasoniae* by *P*. *cosyrae*. This will elucidate the intricate mechanisms underlying host-parasitoid interactions in tephritids and the role of bacterial symbionts in these host-parasitoid bi-trophic models.

Bipartite network analysis revealed occurrence of bacteria genera such as *Pseudomonas*, *Enterobacter*, *Acinetobacter*, *Anoxybacillus*, and *Corynebacterium* which were present across all *B. dorsalis* larvae and the parasitoids, suggesting that these genera represent ubiquitous taxa in both the host and the parasitoids.

The diversity of the fungal community of *B*. *dorsalis* larvae declined significantly as a result of parasitization by either parasitoid species, which further substantiates our argument of parasitoid-induced gut dysbiosis. We postulate that this negative effect of parasitization on *B*. *dorsalis* larval gut mycobiome could be a consequence of parasitoid-induced alteration of host immune responses which inadvertently impact the gut fungal commensals. Alternatively, one could explain the reduced fungal community diversity as a result of the increased relative abundance of some gut bacteria. For example, *S*. *maltophilia* and *Pantoea* species, which were highly abundant in the guts of the parasitized larvae have been shown to inhibit fungal growth [65, 66].

Very few studies have explored the fungal communities of tephritid fruit flies, and even fewer studies [67, 68] have attempted to divulge the roles fungi play in these insects. Nevertheless, available literature suggests that most fungi are essential for nutrient acquisition and host development [67, 69]. While this study presents the first report of *Saccharomyces* species in tephritids, it is not a surprising finding since *Saccharomyces* species like *S. cerevisiae* have been reported in other insect species [70]. Further investigation is warranted to determine its contribution to the eco-physiological functioning of *B. dorsalis*.

In conclusion, our study reveals that different parasitoid species induce distinct changes in insect gut microbial communities. While parasitization by the avirulent *P*. *cosyrae* mainly affected the fungal diversity of *B. dorsalis*, parasitization by the virulent parasitoid, *D*. *longicaudata* altered microbial composition and favored increased relative abundance of pathogenic bacteria, which likely complement its host immune-suppressing arsenals. These findings provide critical insights on the drivers of host-parasitoid interactions and establish a benchmark for further exploration of host-parasitoid-symbiont interactions in frugivorous fruit flies. We also provide baseline information on the mycobiome assemblage of parasitized *B*. *dorsalis*, which presents potential for integration in pest management regimens against this invasive pest. We suggest that future research investigates, using culture-based methods, the influence of the bacterial and fungal communities on the host-parasitoid interactions of *B. dorsalis*.

### Supplementary Information

ESM 1 Online resource 1. Table showing the relative abundance of the bacterial phyla of *B*. *dorsalis* larval guts post-parasitisation by *Diachasmimorpha longicaudata* and *Psyttalia cosyrae*. (XLSX 10.6 kb)

ESM 2 Online resource 2. Table showing the percentage relative abundance of the most common bacterial genera in *Bactrocera dorsalis*. It includes the unparasitised larvae (control) and those parasitised by *Diachasmimorpha longicaudata* (BD-DL) and *Psyttalia cosyra* (BD-PC). (XLSX 11.3 kb)

ESM 3 Online resource 3. Relative abundance of selected bacterial genera across the different *Bactrocera dorsalis* larval groups (Control, parasitized by *Diachasmimorpha longicaudata* and those parasitized by *Psyttalia cosyrae*). (DOCX 333 kb)

ESM 4 Online resource 4. Differential abundance of bacterial ASVs and their taxonomic assignment in *Diachasmimorpha longicaudata* -parasitised *Bactrocera dorsalis* larvae compared to the control. Statistical significance of ASVs is assigned at a p-adjusted (padj) value less than 0.05. (XLSX 71.9 kb)

ESM 5 Online resource 5. Differential abundance of bacterial ASVs and their taxonomic assignment in *Psyttalia cosyrae* -parasitised *Bactrocera dorsalis* larvae compared to the control. Statistical significance of ASVs is assigned at a p-adjusted (padj) value less than 0.05. (XLSX 55.6 kb)

ESM 6 Online resource 6. Differential abundance of bacterial ASVs and their taxonomic assignment in *Psyttalia cosyrae* -parasitised *Bactrocera dorsalis* larvae compared to those parasitised by *Diachasmimorpha longicaudata*. Statistical significance of ASVs is assigned at a p-adjusted (padj) value less than 0.05. (XLSX 80.9 kb)

ESM 7 Online resource 7. Percentage relative abundance of the most common bacteria genera in female *Diachasmimorpha longicaudata* and *Psyttalia cosyrae*. (XLSX 10.9 kb)

ESM 8 Online resource 8. Percentage relative abundance of the most common fungal phyla in *Bactrocera dorsalis*. It includes the unparasitised larvae (control) and those parasitised by *Diachasmimorpha longicaudata* (BD-DL) and *Psyttalia cosyrae* (BD-PC). (XLSX 9.74 kb)

ESM 9 Online resource 9. Percentage relative abundance of the most common fungal genera in *Bactrocera dorsalis*. It includes the unparasitised larvae (control) and those parasitised by *Diachasmimorpha longicaudata* (BD-DL) and *Psyttalia cosyrae* (BD-PC). (XLSX 10.1 kb)

## Data Availability

The datasets generated during and/or analyzed during the current study are available in the Sequence Read Archive (SRA) at NCBI under Bioproject: PRJNA1042921.
