# Supplementary material for: Shaping the Microbial Landscape: Parasitoid-Driven Modifications of Bactrocera dorsalis Microbiota
Source: Microb Ecol. 2024 Jun 3;87(1):81. doi: 10.1007/s00248-024-02393-0 (PMC11147917; doi:10.1007/s00248-024-02393-0)
Supplement: Supplementary file 3 — Online resource 3. Relative abundance of selected bacterial genera across the different Bactrocera dorsalis larval groups (Control, parasitized by Diachasmimorpha longicaudata and those parasitized by Psyttalia cosyrae). (DOCX 333 kb) [file 248_2024_2393_MOESM3_ESM.docx]

Journal: Microbial ecology

**Title: Shaping the microbial landscape: Parasitoid-driven modifications of *Bactrocera* *dorsalis* microbiota**

Authors: Rehemah Gwokyalya^1,2^, Jeremy K. Herren^1^, Christopher W. Weldon^2^, Shepard Ndlela^1^, Joseph Gichuhi^1^, Nehemiah Ongeso^1^, Anne W. Wairimu^1^, Sunday Ekesi^1^, and Samira A. Mohamed^1^

^1^International Centre of Insect Physiology and Ecology; P.O. Box 30772-00100 Nairobi, Kenya

^2^Department of Zoology and Entomology, University of Pretoria, Private Bag X20, Pretoria, South Africa

Correspondence:

Samira A. Mohamed: [sfaris@icipe.org](mailto:sfaris@icipe.org);

Rehemah Gwokyalya: [remysuffy2@gmail.com](mailto:remysuffy2@gmail.com), [rgwokyalya@icipe.org](mailto:rgwokyalya@icipe.org)


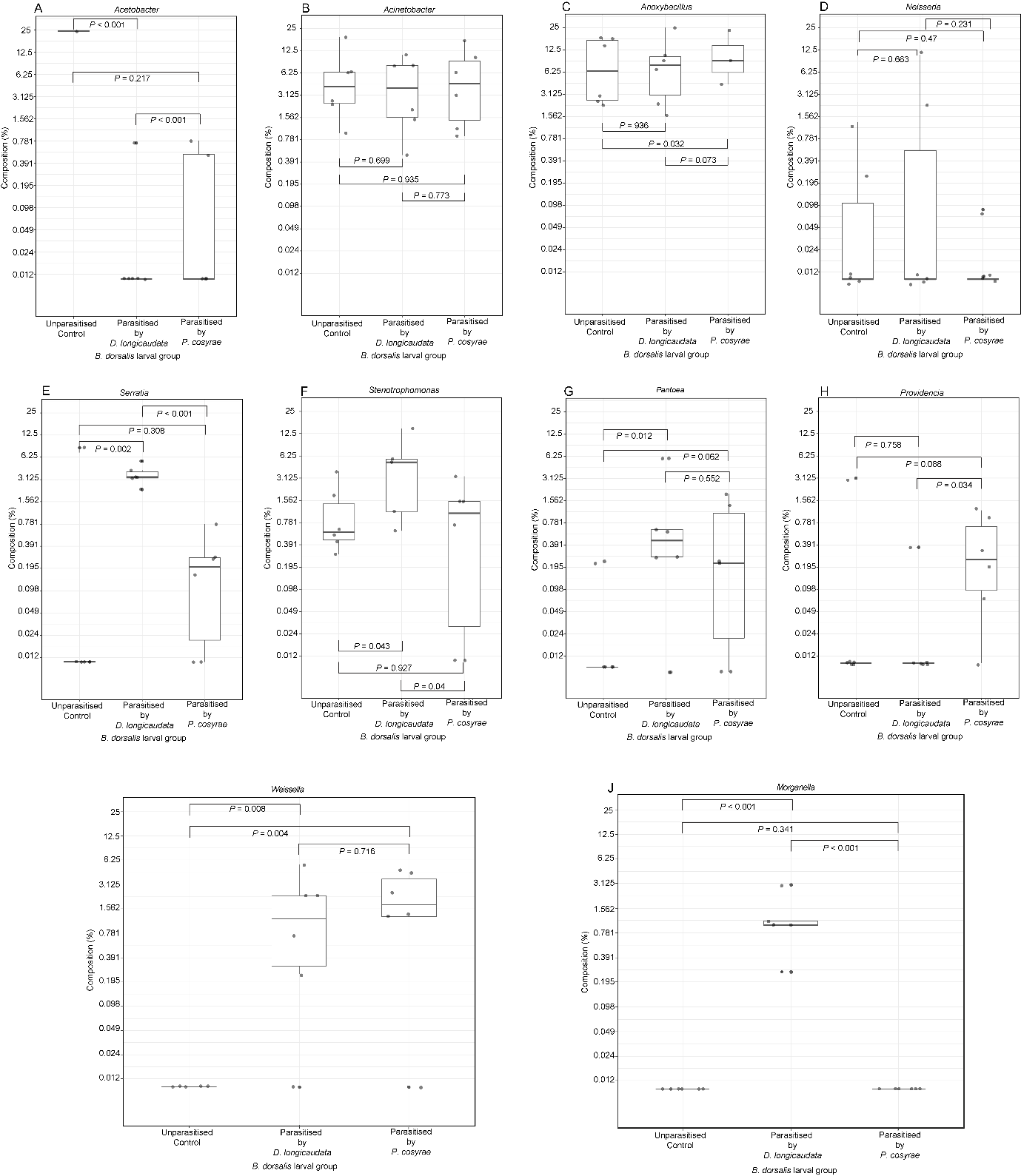


Supplementary figure 1: Relative abundances of selected bacterial genera across the different *Bactrocera* *dorsalis* larval groups (Control, parasitized by *Diachasmimorpha longicaudata* and those parasitized by *Psyttalia cosyrae*). Comparison across groups for A) *Acetobacter*, B) *Acinetobacter* c) *Anoxybacillus*, D) *Neisseria*, E) *Serratia*, F) *Stenotrophomonas*, G) *Pantoea*, H) *Providencia* I) *Weissella* and J) *Morganella*. Comparisons between groups are significant when P < 0.05.
